# Amorphous thin-film oxide power devices operating beyond bulk single-crystal silicon limit

**DOI:** 10.1038/s41598-021-88222-7

**Published:** 2021-05-03

**Authors:** Yuki Tsuruma, Emi Kawashima, Yoshikazu Nagasaki, Takashi Sekiya, Gaku Imamura, Genki Yoshikawa

**Affiliations:** 1 Advanced Technology Research Laboratories, Idemitsu Kosan Co., Ltd., 1280 Kami-izumi, Sodegaura, Chiba 299-0293 Japan; 2 World Premier International Research Center Initiative (WPI), International Center for Materials Nanoarchitectonics (MANA), National Institute for Materials Science (NIMS), 1-1 Namiki, Tsukuba, Ibaraki 305-0044 Japan; 3 Center for Functional Sensor and Actuator (CFSN), National Institute for Materials Science (NIMS), 1-1 Namiki, Tsukuba, Ibaraki 305-0044 Japan; 4 Materials Science and Engineering, Graduate School of Pure and Applied Science, University of Tsukuba, 1-1-1 Tennodai, Tsukuba, Ibaraki 305-8571 Japan; 5 Present Address: Electronic Materials Department, Idemitsu Kosan Co., Ltd., Tokyo, Japan

**Keywords:** Materials science, Condensed-matter physics, Materials for devices

## Abstract

Power devices (PD) are ubiquitous elements of the modern electronics industry that must satisfy the rigorous and diverse demands for robust power conversion systems that are essential for emerging technologies including Internet of Things (IoT), mobile electronics, and wearable devices. However, conventional PDs based on “bulk” and “single-crystal” semiconductors require high temperature (> 1000 °C) fabrication processing and a thick (typically a few tens to 100 μm) drift layer, thereby preventing their applications to compact devices, where PDs must be fabricated on a heat sensitive and flexible substrate. Here we report next-generation PDs based on “thin-films” of “amorphous” oxide semiconductors with the performance exceeding the silicon limit (a theoretical limit for a PD based on bulk single-crystal silicon). The breakthrough was achieved by the creation of an ideal Schottky interface without Fermi-level pinning at the interface, resulting in low specific on-resistance *R*_on,sp_ (< 1 × 10^–4^ Ω cm^2^) and high breakdown voltage *V*_BD_ (~ 100 V). To demonstrate the unprecedented capability of the amorphous thin-film oxide power devices (ATOPs), we successfully fabricated a prototype on a flexible polyimide film, which is not compatible with the fabrication process of bulk single-crystal devices. The ATOP will play a central role in the development of next generation advanced technologies where devices require large area fabrication on flexible substrates and three-dimensional integration.

## Introduction

**Figure 1:**
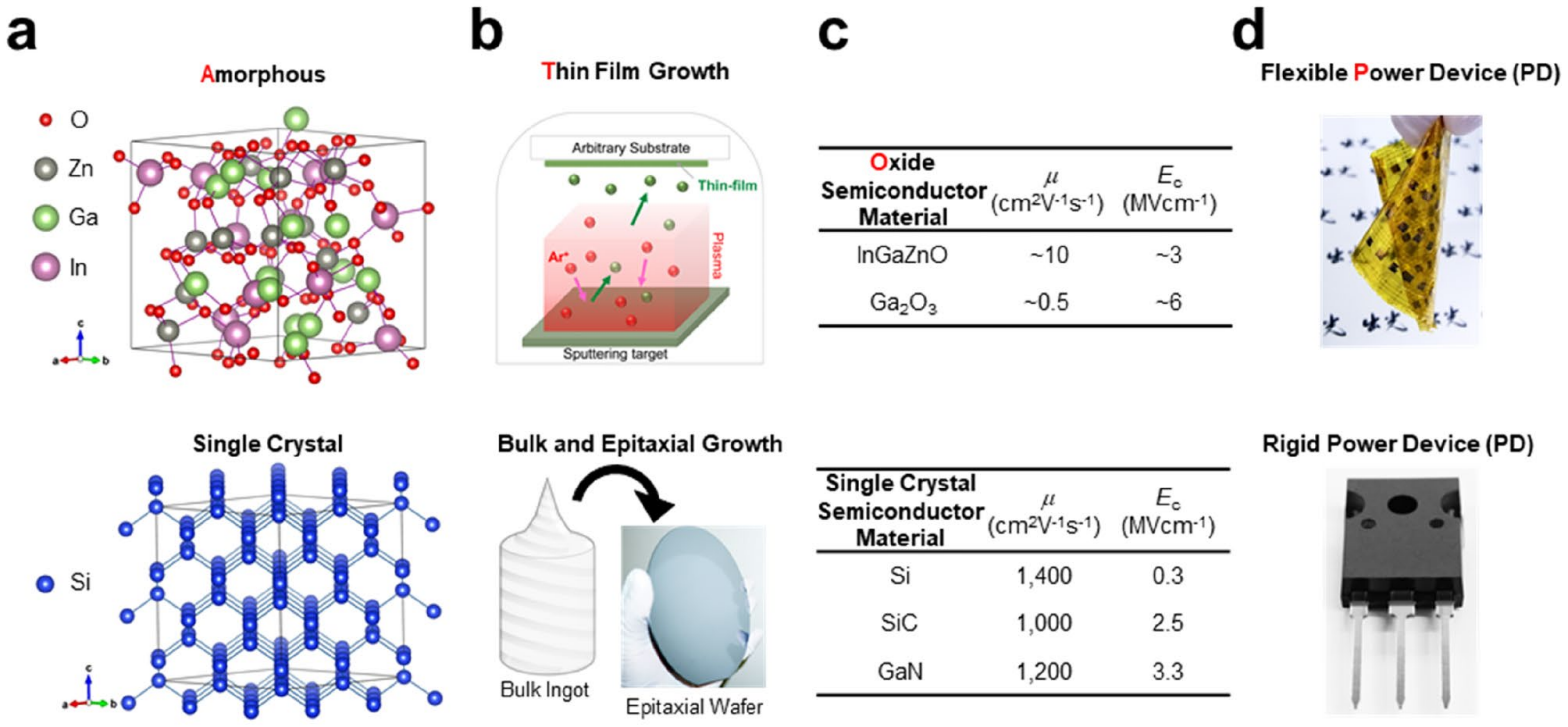
Concepts of amorphous thin-film oxide power device (ATOP, upper panel) compared with conventional bulk single crystal power device (PD, lower panel). (**a**) Structures of amorphous indium–gallium–zinc–oxide (InGaZnO) and single crystal silicon (Si). (**b**) Typical fabrication process of ATOP: thin-film growth on an arbitrary substrate by sputtering. That of the conventional PD: Bulk and epitaxial growth for bulk ingot and single-crystal semiconductor with the bulk substrate, respectively. (**c**) Material parameters of measured mobility *μ* and estimated critical breakdown field *E*_C_ from measured bandgap for amorphous oxide semiconductor materials used in this work, and typical *μ* and *E*_C_ for conventional PD materials. (**d**) PD structures of novel flexible ATOP and conventional rigid discrete.

A power device (PD) is a general term for semiconductor on/off control elements, such as diodes and transistors for energy conversion (e.g. AC–DC conversion)^1–3^. Two important characteristics for designing PDs are specific on-resistance (*R*_on,sp_) and breakdown voltage (*V*_BD_). According to these parameters, the numerical factor related to the efficiency of power conversion, which is critical for PDs, is represented by the figure of merit (FOM) according to the following relationship^1,4^:

1 $${\text{FOM}} = V_{{{{\rm BD}}}}^{2} /R_{{{\rm on,sp}}},$$

which shows that a PD with low energy loss requires a large FOM value. Recently, application of materials such as gallium-nitride (GaN) and silicon-carbide (SiC) are being developed to replace single-crystal silicon (Si) because they are expected to exhibit higher FOM than Si^1,5^. However, PDs fabricated using these materials still suffer from fundamental constraints associated with “bulk” and “single-crystalline” materials that restrict the flexibility of designing devices (Fig. 1 bottom panel)^1,5,6^. Therefore, PDs based on “amorphous” and “thin-film” materials mitigate both of these problems and enable the fabrication of flexible devices using low temperature processes (Fig. 1 top panel). Although there have not been any reports of applications to date, amorphous oxide semiconductors (AOS) typically based on indium–gallium–zinc–oxide (InGaZnO)^7,8^ are candidates for producing PDs because Schottky barrier diodes (SBDs) with low *R*_on,sp_^9–12^ and high *V*_BD_^13,14^ have been reported for these materials, with the high potential of achieving high FOM.

## Results and discussion

**Figure 2:**
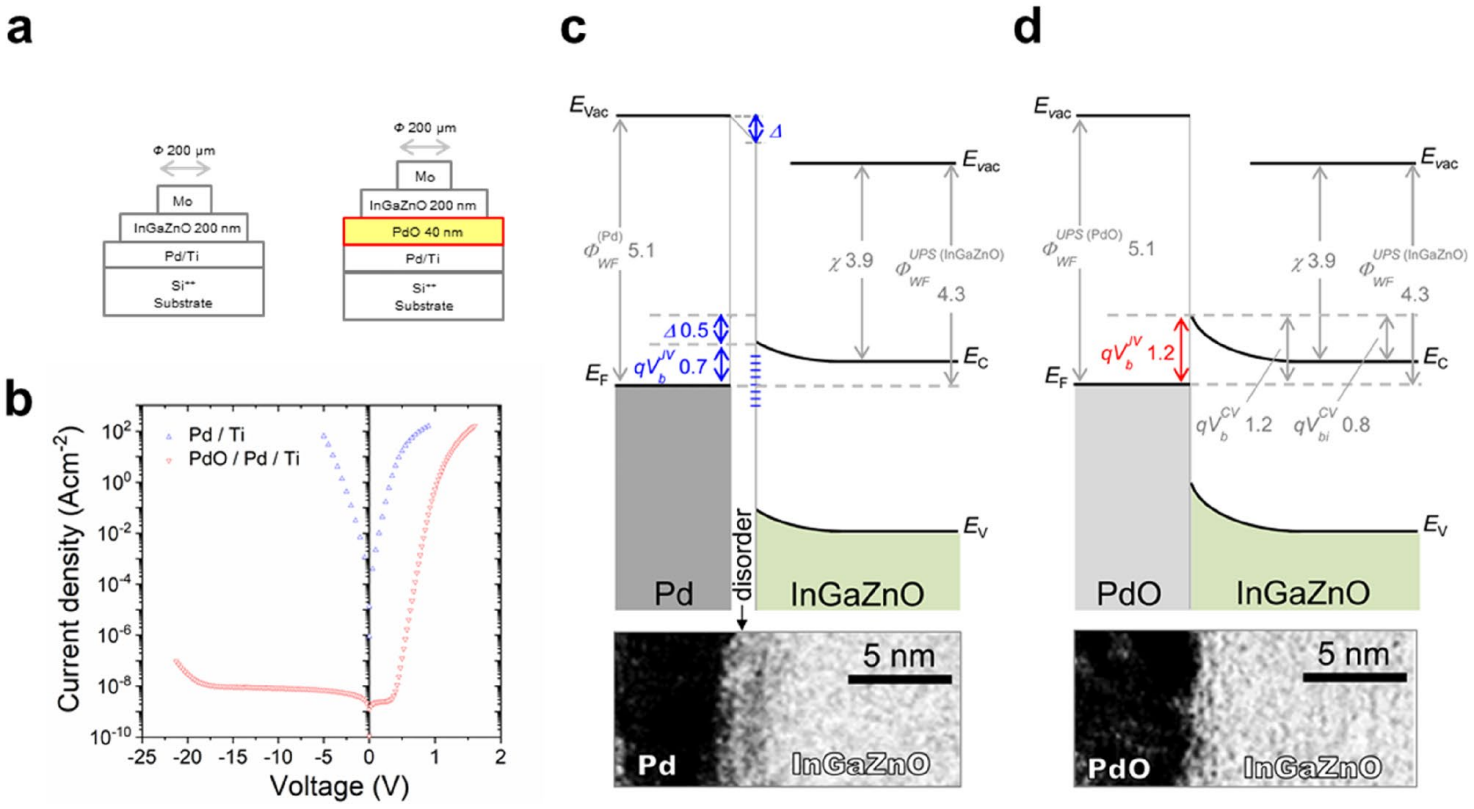
Schottky interface improved by palladium-oxide (PdO) layer for high breakdown voltage *V*_*BD*_. (**a**) Schematic images of Schottky barrier diode (SBD) structures with and without a PdO layer at the Schottky interface. (**b**) Current density (*J*)–voltage (*V*) characteristics of before and after the optimization by PdO layer shown in (**a**). (**c**,**d**) Band diagrams for the Schottky contact of Pd–InGaZnO and PdO–InGaZnO, respectively. The work function *Ф*_WF_, Schottky barrier height *V*_b_ and built-in voltage *V*_bi_ were obtained from the results of UPS (from Supplementary Fig. 2e and Supplementary Table 1b), *J*–*V* (from Supplementary Table 1a) and Capacitance (*C*)–Voltage (*V*) (from Supplementary Table 11c) measurements, respectively. The semiconductor electronic affinity *χ* is determined by *V*_b_^CV^, *V*_bi_^CV^ and *Ф*_WF_^(InGaZnO)^. The interface dipole *Δ* is defined as *Ф*_WF_^(Metal)^ – (*V*_b_^JV^ + *χ*). *Δ* = 0 indicates no pinning effect at the Schottky interface: Pd–InGaZnO contact with pinning (*Δ* ≠ 0) and PdO–InGaZnO contact without pinning (*Δ* = 0). Cross-sectional transmission electron microscope (TEM) images of the interface as shown in lower panel. The disordered interface, which can cause the pinning, is observed in (**c**).

To demonstrate the potential of amorphous thin-film oxide power devices (ATOPs), we first fabricated SBD structures to assess the basic performance of PDs with a view to realizing high FOM. Notably, in order to improve FOM, both low *R*_on,sp_ and high *V*_BD_ have to be achieved at the same time in spite of the trade-off between them^1^. In conventional AOS-SBD, the surfaces of Schottky electrodes of palladium (Pd) are oxidized with UV ozone treatment to improve the Schottky interface for lower *R*_on,sp_ and higher *V*_BD_^13,14^. With this approach, however, it is difficult to achieve high FOM because of a thin ~ 1 nm oxide layer on the Pd surface, resulting in insufficient Schottky interfaces which have relatively high resistance up to ~ 1 × 10^–3^ Ω cm^2^ and low *V*_BD_ up to − 16 V^13^. To resolve this problem, we used a much thicker palladium-oxide (PdO) layer and succeeded in forming PdO layers with arbitrary thicknesses by introducing oxygen-added sputtering, which has not been applied to AOS-SBD so far. As shown in Fig. 2a and b, a 40 nm-thick PdO layer drastically improved *V*_BD_ compared to AOS-SBD without PdO (Supplementary Fig. 1a, Supplementary Table 1a). Although the PdO layer led to an increase in *R*_on,sp_ because of an additional contact resistance, we found that an insertion of a Pd/Titanium(Ti) layer under the PdO layer circumvented this issue, maintaining low *R*_on,sp_ (Supplementary Fig. 1a, Supplementary Table 1a). Furthermore, we also developed a new method to control the drift layer thickness *d*, which is an important parameter as described later to maximize FOM for ATOPs. In contrast to conventional methods^15^, which cannot produce uniform layers with the thicknesses larger than 50 nm, our method enables the formation of uniform layers up to ~ 1000 nm (Supplementary Fig. 2a–c) by adding water vapour during the sputtering (see "Method" section). Accordingly, we succeeded in producing an SBD with *V*_BD_ that varied linearly with *d* (Supplementary Fig. 6, Supplementary Table 1a). Such an optimized SBD was also confirmed to have an ideal Schottky barrier without pinning^16,17^ as shown in Fig. 2c and d.

**Figure 3:**
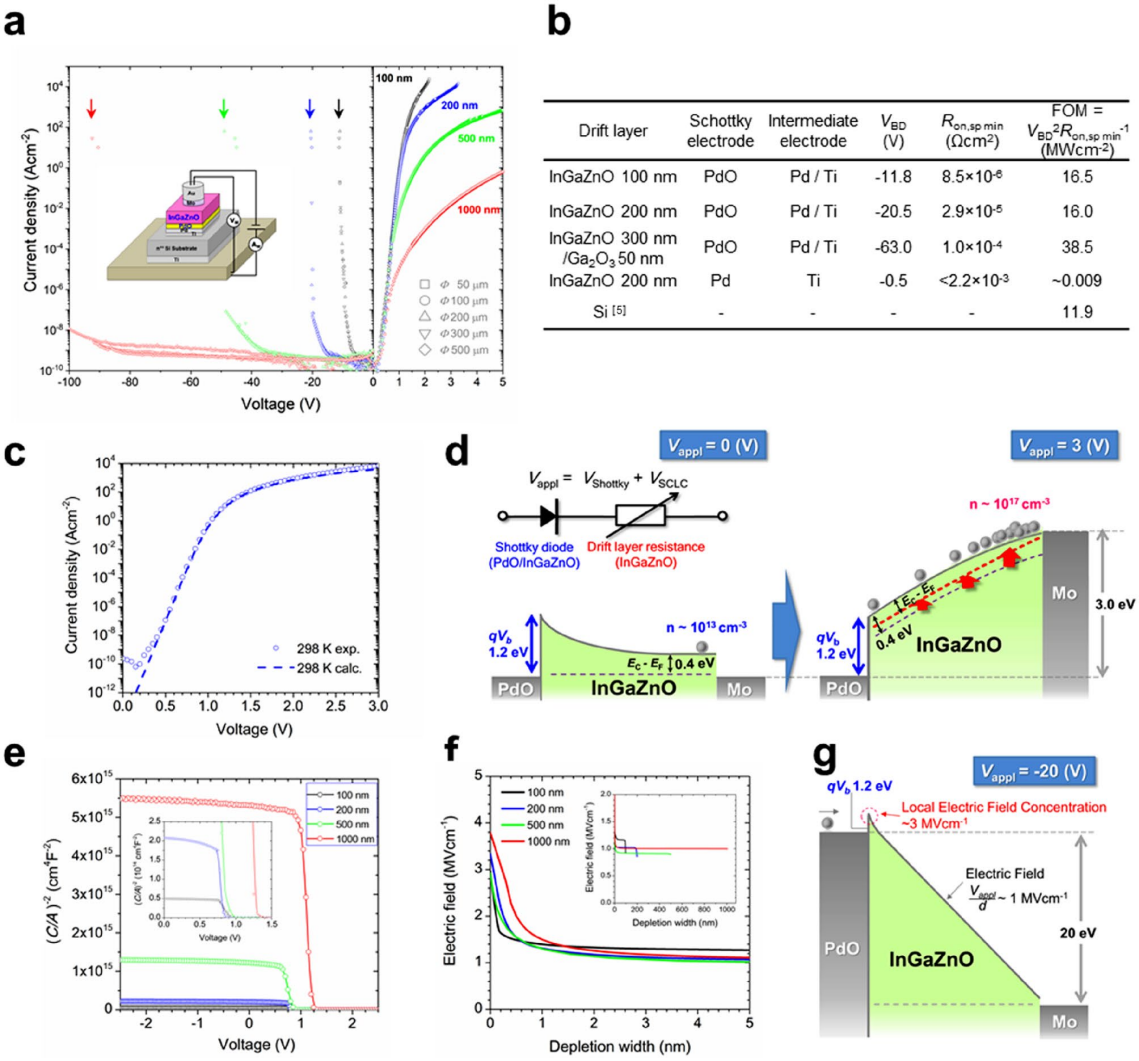
Forward and reverse diode characteristics of ATOP. (**a**) *J–V* characteristics of the ATOP with different drift layer thicknesses. The right and left sides show the forward and reverse characteristics with different scales in horizontal axes, respectively. The thicknesses and the sizes of Au/Mo top electrodes are indicated by different shapes and colours of each data point, respectively. The arrows indicate the breakdown voltage. (**b**) Breakdown voltage *V*_*BD*_ and minimum specific on-resistance *R*_*on,sp min*_ were obtained from *J–V* characteristics in (**a**). Figures-of-Merit (FOM) of power devices were calculated from *V*_*BD*_ and *R*_*on,sp min*_. (**c**) Forward characteristic of 200 nm SBD. The circles and the dashed line show the experimental data and the result of numerical calculation, respectively. The numerical calculation was performed on the basis of the new model taking account of the initial free carrier concentration *n*_*0*_, using Eq. (28) in "Method" section and fitting parameters with Supplementary Table 1d. (**d**) Band diagrams for the forward characteristics in the 200 nm SBD. Left: Common Schottky operation at 0 V. Right: Dominant SCLC operation. The equivalent circuit at the top represents the Schottky-SCLC model with the diode component at the InGaZnO–PdO interface and variable resistance in the drift layer. (**e**) The calculated (C/*A*)^−2^ (the capacitance(*C*) divided by the electrode area(*A*))–voltage(*V*) plots of the ATOPs in (**a**). (**f**) Depth profile of electric fields distribution at the breakdown. (**g**) Breakdown mechanism by the local electrical field concentration at the interface of InGaZnO-PdO at the breakdown voltage of 200 nm SBD.

The schematic illustration of ATOP that we fabricated based on InGaZnO-SBD is shown in Fig. 3a with the current density–voltage (*J*-*V*) dependence on the thickness of each layer and the area of top electrode. The characteristics of the device according to the Schottky theory^18^ were determined from − 5 to + 0.5 V in both forward and reverse directions and were independent of the drift layer thickness and the electrode size. This ATOP exhibited a superior SBD performance with a rectification ratio of over 10^14^, a diode ideal factor (*n*) of approximately 1.1, and a Schottky barrier height (*V*_b_) of 1.2 eV in the forward direction (Supplementary Table 1a). It should be noted that ATOP with 100 nm and 200 nm of *d* exhibited *R*_on,sp_ < 10^–4^ Ω cm^2^ (Fig. 3b), which is difficult to realize even with a single-crystal semiconductor PD^5,19^. In the reverse direction, on the other hand, a flat thermionic emission current characteristic^20^ was observed with less than 10^–9^ A cm^−2^ even under high electric fields before breakdown, which is also challenging even for a single-crystal SBD. The FOMs obtained for these ATOPs are comparable or even higher than theoretical values of single-crystal silicon^5,19,21^, as shown in Fig. 3b.

To clarify the performance limit of ATOP with respect to *R*_on,sp_ and *V*_BD_, we first focused on *R*_on,sp_. For a typical single-crystal PD based on drift conduction, *R*_on,sp_ is given by *d*/*μnq*^1^, where *μ*, *n* and *q* correspond to mobility, carrier density and elementary charge, respectively. It is important to notice that ATOP has a low *R*_on,sp_ irrespective of the low *μ* (~ 10 cm^2^ V^−1^ s^−1^; ~ 1/100 compared to Si in Fig. 1c) of AOSs. Therefore, we constructed a forward conduction model through the ATOP in Fig. 3c and d. As shown in the inset of Fig. 3d, the resistance of an SBD can be regarded as consisting of the two components with the diode at the Schottky interface and the resistance of the drift layer^1^. Notably the latter component is more important for PD design because it is common for a wide range of semiconductor elements. We confirmed that the diode component obeys the relationship based on the common thermionic emission theory through a Schottky contact^18^ (Eq. 5 in "Method" section). As for the resistance of the drift layer, our new model (Eq. 28 in "Method" section) based on the space-charge limited current (SCLC) model^22,23^ with a modification to account for AOS characteristics was found to be applied. It should be noted that the resistance model for the drift layer is not based on conventional drift conduction with single-crystal semiconductors, but on the SCLC conduction, which is current behaviour for the injection of external carriers into substances with low carrier density such as insulators. In the case of ideal solid insulators, the current density is given by *J*_*SCLC, Std.*_ = 9*εμV*^*2*^/8*d*^3^ where *ε* is a dielectric constant; *J*_*SCLC, Std.*_ is inversely proportional to the cube of *d* and proportional to the square of the divided voltage *V* to the drift layer. The characteristics according to the model (Eqs. 5, 7, 28 in "Method" section) were experimentally confirmed in Fig. 3c and also for each temperature and thickness (Supplementary Fig. 3). Furthermore, in the region for applied voltage over 2 V, the resistance of the drift layer was confirmed to obey the ideal SCLC model of *J*_*SCLC, Std.*_ (Supplementary Fig. 4a and b) described above. This behaviour can be ascribed to the reduced effect of trap levels^24^ in the band-gap occupied by electrons supplied from top electrodes (Supplementary Fig. 4c). Figure 3d shows the simplified band diagram in the forward direction of the InGaZnO-SBD for applied voltages of 0 V and 3 V. This diagram shows that the diode component is dominant up to the built-in voltage *V*_*bi*_ of ~ 1.2 V (Fig. 3d left panel) and the SCLC conduction in the drift layer becomes dominant for higher applied voltages (Fig. 3d right panel). Since the ideal SCLC takes place for the low resistance region for applied voltages higher than 2 V in the case of an ATOP (Supplementary Fig. 4d), the *R*_on,sp_ of ATOP can be approximated as follows:

2 $$R_{on,sp.} = \frac{dV}{{dJ}}\sim\frac{{4d^{3} }}{9\varepsilon \mu V}$$

**Figure 4:**
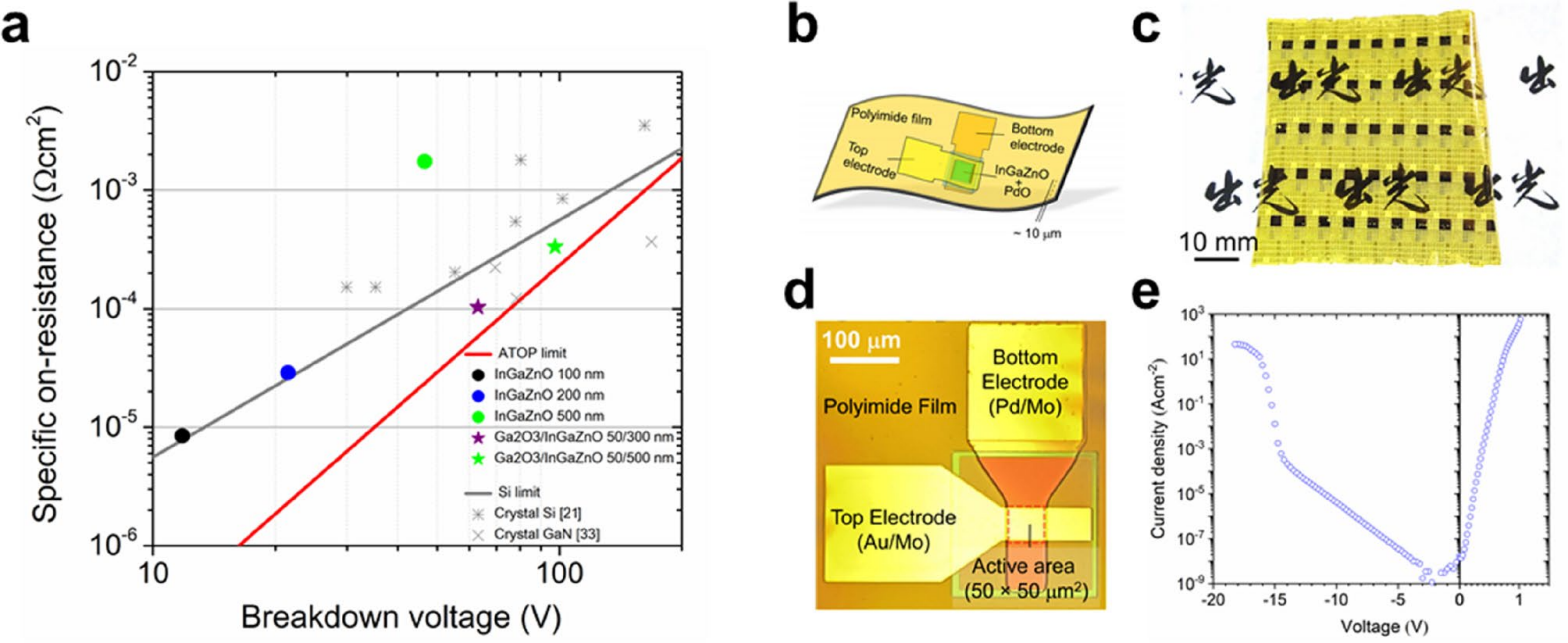
Figure-of-merits (FOM) for PDs and the demonstration of flexible ATOP. (**a**) The relationship between breakdown voltage *V*_*BD*_ and on-specific resistance *R*_*on,sp*_. The circles and the stars correspond to the present results of the single- and double-layer diodes, respectively. The asterisk and cross grey marks are taken from the references to Si and GaN single-crystal semiconductors, respectively. ATOP limit obtained from Eq. (4) with the parameters in Supplementary Fig. 5a and V at 5 V. (**b**) Schematic images of flexible ATOP (**c**) Photograph of ATOPs on polyimide film. (**d**) Photograph of measured SBD on a flexible polyimide film with the optimized diode structure. **e,** *J–V* characteristics of SBD shown in (**d**).

This equation shows that small *d* with large *V* leads to low *R*_on,sp_. In the case of ATOP, *d* is ~ 1/10 smaller than that of bulk single-crystal Si PD, achieving surprisingly low *R*_on,sp_ (< 10^–4^ Ω cm^2^) in spite of low *μ* (~ 1/100), as shown in Supplementary Fig. 5a.

As for the *V*_BD_ that eliminates the effect of layer thickness, we focus on the breakdown electric field *E*_BD_, which is a normalized value of *V*_BD_ divided by *d*. We found that the *E*_BD_ of all the devices we fabricated exhibited around 1 MVcm^−1^ (Supplementary Fig. 6, Supplementary Table 1a). While this value is already higher than that of single-crystal Si (~ 0.3 MVcm^−1^)^5^, it is still lower than the expected value of approximately 3 MVcm^−1^ based on the critical breakdown field *E*_*C*_^25^—intrinsic breakdown field determined by the properties of materials—estimated by the band-gap of InGaZnO, 3.2–3.3 eV (Supplementary Fig. 2f, Supplementary Table 1b). According to the capacitance–voltage (*C*-*V*) measurements and the depth profile of electric fields distribution in Fig. 3e and f (through Supplementary Fig. 7), respectively, it was confirmed that the local electric field concentrating at the PdO–InGaZnO interface (Fig. 3g) caused the breakdown with such a low electric field. It should be noted that the high electric field of 2.9–3.8 MV cm^−1^ in Fig. 3f, comparable to the estimated *E*_*C*_ values, was induced within 1 nm of the interface, resulting in the breakdown of the ATOPs. To achieve higher *E*_BD_, an amorphous gallium-oxide (Ga_2_O_3_) layer having a wider band gap of 4.4 eV (Supplementary Fig. 1c and d, Supplementary Table 1b) was inserted between the PdO and InGaZnO layers. As the results shown in Supplementary Fig. 1c, the *E*_*BD*_ (~ 1.8 MVcm^−1^) was improved to double that of a single PdO layer without sacrificing *R*_on,sp_. This result demonstrates that *E*_BD_ can be improved close to *E*_C_ by optimizing the interface, showing that *V*_BD_ can be designed by the following Eq. (3) for ideal ATOP (Supplementary Fig. 5c):

3 $$V_{{{{\rm BD}}}} \sim E_{{{\rm C}}} \times d$$

Accordingly, the FOM of ATOP can be described by the materials parameters as follows:

4 $${\text{FOM}} = V_{{{{\rm BD}}}}^{2} /R_{{{\rm on,sp}}} \sim 9\varepsilon \mu VE_{c}^{2} /4d.$$

## Methods

### Device fabrication

Heavily doped n^++^ silicon (phosphorus doped, 1 mΩ cm, 300 μm thick, Okmetic) was used as the substrate for the Schottky barrier diodes (SBDs). First, a 150 nm titanium (Ti) film was deposited on the backside of the silicon substrate by DC magnetron sputtering in argon (Ar) atmosphere at 0.5 Pa, as a backside electrode. Next, a 10 nm Ti and a 50 nm palladium (Pd) were deposited on the surface of the substrate under the same sputtering condition as both an adhesion layer and a contact resistance reducing layer. A 40 nm palladium-oxide (PdO) layer, which works as a Schottky electrode, was then deposited by DC magnetron sputtering in oxygen (O_2_) atmosphere at 0.5 Pa. On the PdO layer, thin amorphous indium–gallium–zinc–oxide (InGaZnO) films with different thicknesses (i.e. 100 nm, 200 nm, 500 nm, and 1000 nm) were formed by DC magnetron sputtering of a InGaZnO ceramic target. During the sputtering, a 1% water vapour content (H_2_O/Ar + H_2_O) was introduced under the conditions of 0.5 Pa at room temperature. To form an amorphous gallium-oxide (Ga_2_O_3_) layer, Ga_2_O_3_ target was sputtered under the condition of 0.5 Pa and 1% partial pressure of O_2_. All samples were then annealed at 300 °C for 1 h in an air atmosphere. Finally, 150 nm Mo/ 500 nm gold (Au) was deposited as a top electrode by photolithography. We used different diameters of the electrodes ranging from 50 to 500 µm. To create a flexible device, ~ 10 μm polyimide film was spin-coated on a carrier glass wafer. Then, Mo/Pd layer was deposited as a bottom electrode. For the subsequent processes, the same processes described above were applied. Finally, flexible ATOP with 200 nm InGaZnO is realized by peeling off the polyimide film from the carrier wafer.

### Device evaluation

The current density–voltage (*J*–*V*) characteristics of the diodes were measured with a semiconductor parameter analyser (Agilent B1500 A). Current can be measured over a wide range (from 10^–15^ to 1 A) owing to the several types of equipped SMUs (Source/Measure Unit) and ASU (atto-sense and switch unit). Capacitance–voltage (*C*–*V*) measurements were performed with an Agilent E4980A LCR meter under a frequency condition at 1 kHz. In both the *J*–*V* measurements and the *C*–*V* measurements including temperature dependence, the Ti backside electrode (the anodic electrode) was connected to the stage. Here, a Kelvin (4 probes/wire) connection was used to minimize the measurement error caused by parasitic resistance, that is the residual resistance of the connection cable and contact between the probe and electrode. For voltage measurement and applied current, the two cathodic probes were connected to the top Au electrode of the device, while the two anode cables were connected to the stage. All measurements were performed in a black box with a semi-automatic probe station (Cascade Microtech PA200).

### Diode analysis

#### Forward characteristics

Current density *J* was defined as the current divided by the area of the top electrode. Basic transport properties of a Schottky junction are described by the following equations:

5 $$J = J_{0} \left[ {\exp \left( {\frac{{qV_{Schottky} }}{nkT}} \right) - 1} \right]$$

6 $$J_{0} = A^{**} T^{2} \exp \left( {\frac{{ - q\Phi_{B} }}{kT}} \right)$$

7 $$V_{Schottky} = V - V_{drift}$$

8 $$\Phi_{B} = - \frac{kT}{q}\ln \left( {\frac{{J_{0} }}{{A^{**} T^{2} }}} \right)$$

9 $$n = \frac{q}{kT}\left( {\frac{dV}{{d\ln (J)}}} \right)$$

10 $$\ln \left( {\frac{{J_{0} }}{{T^{2} }}} \right) = \ln (A^{**} ) - \frac{{q\Phi_{B.M} }}{kT}$$

where *V*_Schottky_ is the voltage applied to the Schottky interface, *n* is the ideality factor (*n* = 1 is the ideal value), *Φ*_B_ is the height of the Schottky barrier, *k* is the Boltzmann constant, *q* is the elementary charge, *T* is absolute temperature and *V* is the applied voltage to the device. Equation (6) shows the saturation current density *J*_0_ and is equivalent to the intercept of ln*J* in (5)^18^. Equation (7) shows the relationship between *V*_Schottky_, *V* and *V*_drift_ (*V*_drift_ is the voltage distributed to the semiconductor drift layer)^1,26^. Equations (8) and (9) are derived from Eqs. (6) and (5), respectively. A** is the effective Richardson constant; the theoretical value for InGaZnO is 41 A cm^−2^ K^−2^ (calculated from m* = 0.34 *m*_*e*_)^27^. In this paper, *Φ*_B_ and *n* were calculated for *T* = 298 K and A** = 42 A cm^−2^ K^−2^. *Φ*_B_ and A** were evaluated by the Richardson plot (Supplementary Fig. 8b). As shown in Eq. (10), the mean barrier height *Φ*_B.M_, which is independent of A** and *T*, is obtained by plotting *J*_0_ against temperature *T.* Experimental results obtained with the 200 nm InGaZnO SBD was analysed according to Eq. (10), resulting in A** = 42 Acm^−2^ K^−2^ and *Φ*_B.M_ = *Φ*_B_.

#### Reverse characteristics

Breakdown voltage *V*_*BD*_ is defined as the highest voltage that is recorded just before a current continuously exceeds 1 μA for more than three measurement points. In this study, it was found that the depletion width *W*_*D*_ is approximately the same as the film thickness *d* of the oxide semiconductor when a reverse voltage (negative bias) is applied (Supplementary Fig. 7a, Supplementary Table 1c). Therefore, it is reasonable to assume that the electric field is homogeneously distributed in the semiconductor drift layer. The breakdown strength *E*_BD_ was defined as the breakdown voltage *V*_*BD*_ divided by the film thickness *d* as the following form:

11 $$E_{BD} = \frac{{V_{BD} }}{d}$$

#### CV measurements

From the measured capacitance of the diode *C*, the depletion width *W*_*D*_ at different voltages, the built-in voltage *V*_*bi*_, and the charge density which contributes to the depletion layer *N*_*delp*_, were obtained according to the following equations^18^:

12 $$W_{D} = \frac{{\varepsilon_{0} \varepsilon_{r} }}{C}A$$

13 $$\frac{{A^{2} }}{{C^{2} }} = \left( {\frac{2}{{\varepsilon_{0} \varepsilon_{r} N_{depl} }}} \right)\left( {V_{bi} - V - \frac{kT}{q}} \right)$$

where *ε*_*r*_ is the static dielectric constant of the semiconductor, *ε*_*0*_ is the dielectric constant of a vacuum and *A* is the active area of the SBD. The Schottky barrier height *Φ*_*B,CV*_ can be calculated from the result of *C*–*V* measurements according to the following equation:

14 $$\Phi_{B,CV} = V_{bi} + \frac{kT}{q}\ln \left( {\frac{{N_{c} }}{{N_{e} }}} \right)$$

where *N*_*C*_ is the effective density of state in the conduction band. In the case of InGaZnO, *N*_*C*_ is 5.2 × 10^18^ cm^−3 ^^28^. *N*_*e*_ is the free charge density, which can be experimentally obtained by Hall measurements.

#### Power law

The forward *J*–*V* characteristics can be described as the following equation:

15 $$J = KV^{m}$$

where *K* is a constant and *m* is the power law index. By differentiating the logarithm of both sides of Eq. (15), *m* is obtained the following equation:

16 $$m = \frac{d\log J}{{d\log V}}$$

Thus, *m* depends on *V* in the SCLC conduction according to the *J*–*V* relationship described as Eqs. (17) and (18). Based on this Eq. (16), we discussed various conduction models [Eqs. (17), (18), and (28)] and the drift model (*J*_ohmic_ = *μnq V/d*) in Supplementary Fig. 4b.

#### Existing SCLC model

The space charge limited current (SCLC) between two terminals forming Ohmic contacts can be explained by the Child’s law for solids. In the simplest standard model, the relationship between current density *J*_SCLC,Std_ and applied voltage *V* can be given as the following equation^22^:

17 $$J_{SCLC,Std.} = \frac{{9\varepsilon \mu V^{2} }}{{8d^{3} }}$$

where *ε* is the permittivity of the semiconductor, *μ* is the carrier mobility and *d* is the film thickness. When the carriers are affected by exponentially distributed traps (EDTs), the current changes according to the filling of traps below the quasi-Fermi level. Assuming the traps with an exponential energy distribution in the bandgap, the current *J*_SCLC,EDT_ can be described by the following equation^23^:

18 $$J_{SCLC,EDT} = N_{c} \mu q\left( {\frac{\varepsilon }{{qN_{t} }}} \right)^{l} \left( {\frac{l}{l + 1}} \right)^{l} \left( {\frac{2l + 1}{{l + 1}}} \right)^{l + 1} \frac{{V^{l + 1} }}{{d^{2l + 1} }}$$

where *N*_*c*_ is the effective density of states, *N*_*t*_ is the total number of traps per unit volume. *l* is defined as *l* = *T*_*t*_/*T*, where *T*_*t*_ is the characteristic temperature which determines the trap distribution. The validity of this model is supported by the result of deep-level transient spectroscopy (DLTS), which shows the distribution of the subgap states in the amorphous oxide semiconductor^24^. However, the effect of the free carrier concentration at the steady state was not considered in this model. Based on our model validation results, Eq. (18) needs to be modified so as to include the effect of initial free carrier concentration. Therefore, referring to Reference^29^, we expanded (18) to include the effect of the initial free carrier concentration.

#### New SCLC model

We modified Eq. (18) so as to include the effect of initial free carrier concentration *n*_0_. According to the reference^29^, the current considering *J*_*SCLC,EDT,n0*_ can be described as follows:

19 $$\begin{gathered} \hfill \\ J_{{_{SCLC,EDT,n0} }} = q\mu n(x)E(x) = q\mu \left[ {n_{0} + n_{i,f} (x)} \right]E(x) \hfill \\ \end{gathered}$$

20 $$\frac{dE}{{dx}} = \frac{q}{\varepsilon }n_{inj} (x) = \frac{q}{\varepsilon }\left[ {n_{i,f} (x) + n_{i,t} (x)} \right]$$

21 $$d = \int_{0}^{d} {dx} = \int_{0}^{{E_{d} }} {\frac{dx}{{dE}}dE}$$

22 $$V_{{_{SCLC} }} = \int_{0}^{d} {E(x)dx} = \int_{0}^{{E_{d} }} {E\frac{dx}{{dE}}dE}$$

where *n*(*x*) is the the carrier concentration contributing to conduction (*x* = 0 is the edge of the top electrode), *n*_*i,f*_(*x*) is the concentration of untrapped injected free carriers, *n*_*i,t*_(*x*) is the concentration of trapped injected carriers, and *n*_*inj*_(*x*) is the total concentration of injected carriers. Equations (19) and (20) show the drift current and Poisson’s equation, respectively. Equation (28) can be derived from Eqs. (19) and (20). Equations (21) and (22) show the boundary conditions. The diffusion current is not considered as its effect can be negligible in the present current region.

First, the exponential trap distribution *n*_*t*_(ε) is defined by the following equation:

23 $$n_{t} ({\upvarepsilon }) \equiv \, \frac{{N_{t} }}{{kT_{t} }}e^{{\left( {{{\upvarepsilon - \upvarepsilon }}_{{{\rm c}}} } \right)/kT_{t} }}$$

where ε is the energy level of the trap states.

Using the Fermi–Dirac distribution as a step function, *n*_*i,t*_(*x*) can be written as shown in the following equation:

24 $$n_{i,t} (x) \equiv \int_{{F_{0} }}^{F(x)} {n_{t} ({\upvarepsilon }) \, d} {\upvarepsilon } = N_{t} \left[ {e^{{\left( {F(x){{ - \upvarepsilon }}_{{{\rm c}}} } \right)/kT_{t} }} - e^{{\left( {F_{0} {{ - \upvarepsilon }}_{{{\rm c}}} } \right)/kT_{t} }} } \right]$$

where *F*_*0*_ is the Fermi level in thermal equilibrium. As the quasi-Fermi level *F*(*x*) shows the depth dependence, the concentration of trapped injected electrons at a position *x* can be estimated from Eq. (24). Since the concentration of the injected electrons is high near the cathode, the position of the quasi-Fermi level is also relatively closer to the conduction band in that region. To connect *n*_*i,t*_(*x*) with the concentration of total electrons that contribute to conduction, we rearrange Eq. (24) by using the Boltzmann’s distribution law:

25 $$n_{i,t} (x) = N_{t} \left[ {\left( {\frac{n(x)}{{N_{c} }}} \right)^{{T/T_{t} }} - \left( {\frac{{n_{0} }}{{N_{c} }}} \right)^{{T/T_{t} }} } \right]$$

Equation (25) shows the trap distribution including the effect of the initial free carrier concentration. Based on Eq. (25) and the Poisson’s Eq. (20), the following equation can be derived:

26 $$\frac{dE}{{dx}} = \frac{q}{\varepsilon }n_{inj} (x) = \frac{q}{\varepsilon }\left\{ {n(x) - n_{0} + N_{t} \left[ {\left( {\frac{n(x)}{{N_{c} }}} \right)^{{T/T_{t} }} - \left( {\frac{{n_{0} }}{{N_{c} }}} \right)^{{T/T_{t} }} } \right]} \right\}$$

From Eqs. (19), (26) can be written as

27 $$\frac{dE}{{dx}} = \frac{q}{\varepsilon }\left\{ {\frac{{J_{SCLC,EDT,n0} }}{q\mu E(x)} - n_{0} + N_{t} \left[ {\left( {\frac{{J_{SCLC,EDT,n0} }}{{q\mu E(x)N_{c} }}} \right)^{{T/T_{t} }} - \left( {\frac{{n_{0} }}{{N_{c} }}} \right)^{{T/T_{t} }} } \right]} \right\}$$

By integrating Eq. (27), the voltage applied to the drift layer *V*_*SCLC*_ can be obtained, which represents a new SCLC model considering the effect of the initial free carrier concentration.

28 $$V_{{_{SCLC} }} = \frac{\varepsilon }{q} \int\limits_0^{E_d} {\frac{E}{{\frac{{J_{{_{{SCLC,EDT,n_{0} }} }} }}{{q\mu E}} - n_{{_{0} }} + N_{t} \left[ {\left( {\frac{{J_{{_{{SCLC,EDT,n0}} }} }}{{q\mu EN_{c} }}} \right)^{{T/T_{t} }} - \left( {\frac{{n_{{_{0} }} }}{{N_{c} }}} \right)^{{T/T_{t} }} } \right]}}} dE$$

To determine *E*_d_ (a numerically calculated value), the boundary condition is written as follows:

29 $$d = \frac{\varepsilon }{q} \int\limits_0^{E_d} {\frac{1}{{\frac{{J_{{_{{SCLC,EDT,n_{0} }} }} }}{{q\mu E}} - n_{{_{0} }} + N_{t} \left[ {\left( {\frac{{J_{{_{{SCLC,EDT,n0}} }} }}{{q\mu EN_{c} }}} \right)^{{T/T_{t} }} - \left( {\frac{{n_{{_{0} }} }}{{N_{c} }}} \right)^{{T/T_{t} }} } \right]}}} dE$$

Here, *E*(0) is set at 0. Note that Eqs. (28) and (29) must be calculated under the following conditions: *J*_*SCLC,EDT,n0*_ , *V*_*SCLC*_ ≥ 0 and *E(x), E*_*d*_ ≥ 0. By using (28), it becomes possible to discuss the mechanism of EDT-type SCLC conduction, in which the concentration of free carriers at the steady state is considered.

To explain the experimentally-obtained *J–V* characteristics, we performed numerical calculations as the current density components of Eqs. (5) and (28) coincide with an intervening variable parameter of *V*_*SCLC*_. In the new SCLC model described as Eq. (28), we treated *J*_*SCLC,EDT,n0*_ as an arbitrary input parameter. *μ* and *n*_0_ are obtained through the Hall measurement. *T*_*t*_ and *N*_*t*_ are fitting parameters to obtain *V*_*SCLC*_. The sample temperature was used for *T,* and *ε* was set at the converted value in the case of *ε*_*r*_ = 16 (determined from the *C-V* measurements). By verifying *J* in Eq. (5) corresponding to the arbitrary *J*_*SCLC,EDT,n0*_ in Eq. (28), comparison between the experimental results and the numerical calculation results can be possible. As *J* and *J*_*SCLC,EDT,n0*_ should be the same value for the series circuit model as shown is Fig. 3d, *V*_*drift*_ in Eq. (7) can be replaced by *V*_*SCLC*_ in Eq. (28). Based on the results of the temperature dependence (Supplementary Fig. 8), *Φ*_*B*_ and *n* in Eq. (5) are determined from Eqs. (8) and (9), respectively. Then, we can obtain *J* from Eq. (5) through *V*_*Schottky*_ as *V* – *V*_SCLC_. In the case of *J* = *J*_*SCLC,EDT,n0*_, the fitting parameter of *T*_*t*_ and *N*_*t*_ are correct.

#### Estimation of ***E***_c_

The critical breakdown field *E*_c_ (the maximum electric field at the Schottky interface when the breakdown voltage *V*_BD_ is applied) can be estimated according to the relationship between the depletion layer width *W*_*D*_ and the charge concentration contributing to the depletion layer *N*_*depl*_ as shown in Eqs. (12) and (13). The calculations were performed with a model that considers the charge density distribution of a one-sided abrupt junction^30^. We assumed that the effect of minority carriers on the space charge formation is negligible. By using the depth profile of *N*_*depl*_ obtained from the *C*–*V* measurements, the field distribution in the film thickness direction can be calculated by the following equation:

30 $$\frac{dE}{{dx}} = - \frac{q}{\varepsilon }N_{depl}$$

As the reverse current is almost 0, Eq. (30) can be written as the following form:

31 $$E(x) = - \frac{q}{\varepsilon }\int_{0}^{x} {\,\,\,\,N_{depl} \,\,dx\,\,} + E(0)$$

By integrating Eq. (31), *V* can be described as

32 $$V = - \frac{q}{\varepsilon }\int_{0}^{{W_{D} }} {\,\,\,\left( {\,\int_{0}^{x} {N_{depl} \,\,dx} } \right)\,dx\,} + E(0)W_{D}$$

where *E(x)* is the electric field intensity at a position *x*, and *E*(0) is the electric field at the Schottky interface. *W*_*D*_can be approximated to *d* as the depletion layer is formed through the whole diode even at 0 V. From the definition of *E*_c_, *E*(0) can be replaced by *E*_c_ when the breakdown voltage *V*_*BD*_ is applied. Then, Eq. (32) can be written as the following form:

33 $$V_{BD} = - \frac{q}{\varepsilon }\int_{0}^{{W_{D} }} {\,\,\,\left( {\,\int_{0}^{x} {N_{depl} \,\,dx} } \right)\,dx\,} + E_{C} W_{D}$$

*E*_c_ can be estimated from Eq. (33) by using the profiling of *E* as described in Eq. (31). *V*_*BD*_ and the *N*_*depl*_ profile for *W*_*D*_ can be experimentally obtained from the *J*–*V* characteristics and the results of the *C*–*V* measurements. The resolution of this technique depends on the Debye screening length; that is, the length by the influence of the impurity concentration in the semiconductor. Thus, the resolution is better than 1 nm for *N*_*depl*_ > 10^19^ cm^−3^ at the Schottky interface^31,32^.

## Characterization of thin film

Cross-sectional observation of the SBDs by transmission electron microscopy (TEM) was performed with a focused-ion-beam device (FB-2100, Hitachi) and TEM (JEM-2800, JEOL). TEM observations were performed at an accelerating voltage of 200 keV. X-ray diffraction (XRD) was performed with a Rigaku SmartLab system under a condition of 40 kV and 40 mA by using the Cu–Kα line (l.5406 Å). The same XRD system was also used to evaluate the crystallinity of the thin films: the grazing incident X-ray diffraction (GIXD, angle of incidence ω) and the conventional θ − 2θ scan (XRD). The thin film samples were deposited on a quartz glass substrate for the XRD measurements and annealed at 300 °C for 1 h before the measurement. The GIXD measurement was performed for a multilayer sample of PdO thin films to avoid the change in crystallinity by annealing on a different substrate. This analysis was also done in the multilayer condition of the actual diode structure to avoid transformation of the polycrystalline structure of the PdO during annealing on a different substrate.

To estimate the bandgap *E*_g_, UV–VIS spectroscopy (V-370, JASCO) was performed for the thin film samples deposited on quartz glass substrates. The samples were annealed before the measurement. The work function *Φ*_WF_ was determined by using ultraviolet photoelectron spectroscopy (UPS) by focusing on the secondary electron cutoff. Monolayer thin films deposited on a Si substrate were used as samples. In the UPS measurements, monochromatized 7.7 eV photons from a D2 lamp were used, and a negative bias of 10 V was applied to the sample. A customized system equipped with a zero-dispersion type double monochromator and a 120 mm hemispherical analyser (PSP Vacuum Technology RESOLVE120) was used for the measurement.

Hall mobility *μ*_Hall_, the free electron carrier concentration *n*_Hall_ and the specific electrical resistance *ρ* were obtained with a Hall measurement system (ResiTest 8400, TOYO Corporation) by using an AC magnetic field. The measurements were performed with the Van der Pauw method at room temperature. Thin films deposited on quartz glass substrates (10 mm × 10 mm) were used as samples. The samples were annealed before the measurement, and indium electrodes were deposited on the 4 corners of the samples.

## Conclusions

The properties of the ATOPs fabricated in this study are summarized in Fig. 4a, comparing with conventional PDs. The theoretical limit of single-crystal Si can be estimated by ${\text{FOM}} = \varepsilon \mu E_{c}^{3} /4$ through materials parameters^1,4^. For the ATOP, on the other hand, a superior performance to single-crystal Si was confirmed for both experimental values and the theoretical limit estimated by Eq. (4). It is worth noting that the ATOP exhibits higher performance than GaN transistors^33^, which have the advantage in lowering resistance, in the region with *V*_BD_ < 100 V and *R*_on,sp_ < 10^–4^ Ω cm^2^. This ultra-low resistance is one of the advantages of ATOP, which has almost no restriction on the type of substrate and is not significantly affected by the contact resistance unlike that of the conventional PDs. Finally, we demonstrated the fabrication of devices on a flexible polyimide film (Fig. 4b–e), making most of the characteristics of ATOP that enables low temperature (≤ 300 °C) sputtering on any type of substrate. ATOP has the potential to replace conventional bulk single-crystal PDs, thereby expanding the applications of PDs into new areas, where bulk single-crystal is intrinsically unable to reach.

## Supplementary Information

Supplementary Figures and Tables

## References

[1] Baliga J (2008). Fundamentals of Power Semiconductor Devices.

[2] Jeon S (2012). High performance bilayer oxide transistor for gate driver circuitry implemented on power electronic devices. Symp. VLSI Technol. Dig..

[3] Shenai K (2013). Switching MegaWatts with power transistors. J. Electrochem. Soc..

[4] Kondrath N, Kazimierczuk MK (2010). Characteristics and applications of silicon carbide power devices in power electronics. INTL J. Electron. Telecommun..

[5] Okumura H (2006). Present status and future prospect of widegap semiconductor high-power devices. Jpn. J. Appl. Phys..

[6] Momma K, Izumi F (2011). VESTA 3 for three-dimensional visualization of crystal, volumetric and morphology data. J. Appl. Crystallogr. A.

[7] Nomura K (2004). Room-temperature fabrication of transparent flexible thin-film transistors using amorphous oxide semiconductors. Nature.

[8] Kamiya T, Nomura K, Hosono H (2010). Present status of amorphous In–Ga–Zn–O thin-film transistors. Sci. Technol. Adv. Mater..

[9] Lee, M. J. *et al.* 2-stack 1D-1R cross-point structure with oxide diodes as switch elements for high density resistance RAM applications, in *Proceedings of the IEEE International Electron Devices Meeting,* 771–777 (2007)

[10] Lee DH, Nomura K, Kamiya T, Hosono H (2011). Diffusion-limited a-IGZO/Pt Schottky junction fabricated at 200 °C on a flexible substrate. IEEE Elec. Dev. Lett..

[11] Chasin A (2014). High-performance a-IGZO thin film diode as selector for cross-point memory application. IEEE Electron Device Lett..

[12] Zhang J (2015). Flexible indium–gallium–zinc–oxide Schottky diode operating beyond 2.45 GHz. Nat. Commun..

[13] Chasin A (2012). High-performance a-In–Ga–Zn–O Schottky diode with oxygen-treated metal contacts. Appl. Phys. Lett..

[14] Xin QH, Yan L, Luo Y, Song A (2015). Study of breakdown voltage of indium–gallium–zinc–oxide-based Schottky diode. Appl. Phys. Lett..

[15] Nomura K, Kamiya T, Hosono H (2013). Effects of diffusion of hydrogen and oxygen on electrical properties of amorphous oxide semiconductor, In–Ga–Zn–O. ECS J. Solid State Sci. Technol..

[16] Léonard F, Tersoff J (2000). Role of fermi-level pinning in nanotube Schottky diodes. Phys. Rev. Lett..

[17] Brillson LJ, Lu Y (2011). ZnO Schottky barriers and Ohmic contacts. J. Appl. Phys..

[18] Rhoderick EH, Williams RH (1988). Metal-Semiconductor Contacts.

[19] Wang Z, Zhang B, Fu Q, Xie G, Li Z (2012). An L-shaped trench SOI-LDMOS with verticaland lateral dielectric field enhancement. IEEE Elec. Dev. Lett..

[20] Hatakeyama T, Shinohe T (2002). Reverse characteristics of a 4H–SiC Schottky barrier diode. Mater. Sci. Forum.

[21] Shenai K (1990). Optimally scaled low-voltage vertical power MOSFET's for high-frequency power conversion. IEEE Transact. Elec. Dev..

[22] Lampert MA, Mark P (1970). Current Injection in Solids.

[23] Mark P, Helfrich W (1962). Space-charge-limited currents in organic crystals. J. Appl. Phys..

[24] Chasin A (2014). Deep-level transient spectroscopy on an amorphous InGaZnO_4_ Schottky diode. Appl. Phys. Lett..

[25] Higashiwaki M, Sasaki K, Kuramata A, Masui T, Yamakoshi S (2012). Gallium oxide (Ga_2_O_3_) metal-semiconductor field-effect transistors on single-crystal β-Ga_2_O_3_ (010) substrates. Appl. Phys. Lett..

[26] Cibils RM, Buitrago RH (1985). Forward I–V plot for nonideal Schottky diodes with high series resistance. J. Appl. Phys..

[27] Takagi A (2005). Carrier transport and electronic structure in amorphous oxide semiconductor, a-InGaZnO_4_. Thin Solid Films.

[28] Kamiya T, Nomura K, Hosono H (2009). Origins of high mobility and low operation voltage of amorphous oxide TFTs: electronic structure, electron transport, defects and doping. J. Disp. Technol..

[29] Chen CW, Wu CI (2008). Analytical solution to space charge limited currents with exponentially distributed traps. J. Appl. Phys..

[30] Glover GH, Tantraporn W (1975). Doping profile measurements from avalanche space−charge resistance: a new technique. J. Appl. Phys..

[31] Kennedy DP, Murley PC, Kleinfelder W (1968). On the measurement of impurity atom distributions in silicon by the differential capacitance technique. IBM J. Res. Dev..

[32] Schubert EF (1990). Spatial resolution of the capacitance-voltage profiling technique on semiconductors with quantum confinement. Appl. Phys. Lett..

[33] Roccaforte F (2014). Challenges for energy efficient wide band gap semiconductor power devices. Phys. Status Solidi A.

