# Supplementary material for: Amorphous thin-film oxide power devices operating beyond bulk single-crystal silicon limit
Source: Sci Rep. 2021 May 3;11:9435. doi: 10.1038/s41598-021-88222-7 (PMC8093298; doi:10.1038/s41598-021-88222-7)
Supplement: Supplementary file 1 — Supplementary Figures and Tables [file 41598_2021_88222_MOESM1_ESM.docx]

**Supplementary Information: Amorphous thin-film oxide power devices operating beyond bulk single-crystal silicon limit**

Yuki Tsuruma^1^†*, Emi Kawashima^1^†, Yoshikazu Nagasaki^1^†, Takashi Sekiya^1^, Gaku Imamura^2,3^, Genki Yoshikawa^2,3,4^

^1^Advanced Technology Research Laboratories, Idemitsu Kosan Co., Ltd., 1280 Kami-izumi, Sodegaura, Chiba 299-0293, Japan

^2^World Premier International Research Center Initiative (WPI), International Center for Materials Nanoarchitectonics (MANA), National Institute for Materials Science (NIMS), 1-1 Namiki, Tsukuba, Ibaraki 305-0044, Japan

^3^Center for Functional Sensor & Actuator (CFSN), National Institute for Materials Science (NIMS), 1-1 Namiki, Tsukuba, Ibaraki 305-0044, Japan

^4^Materials Science and Engineering, Graduate School of Pure and Applied Science, University of Tsukuba, 1-1-1 Tennodai, Tsukuba, Ibaraki, 305-8571, Japan

*Correspondence to: yuki.tsuruma.2170@idemitsu.com

†Present address: Electronic Materials Department, Idemitsu Kosan Co., Ltd.

**
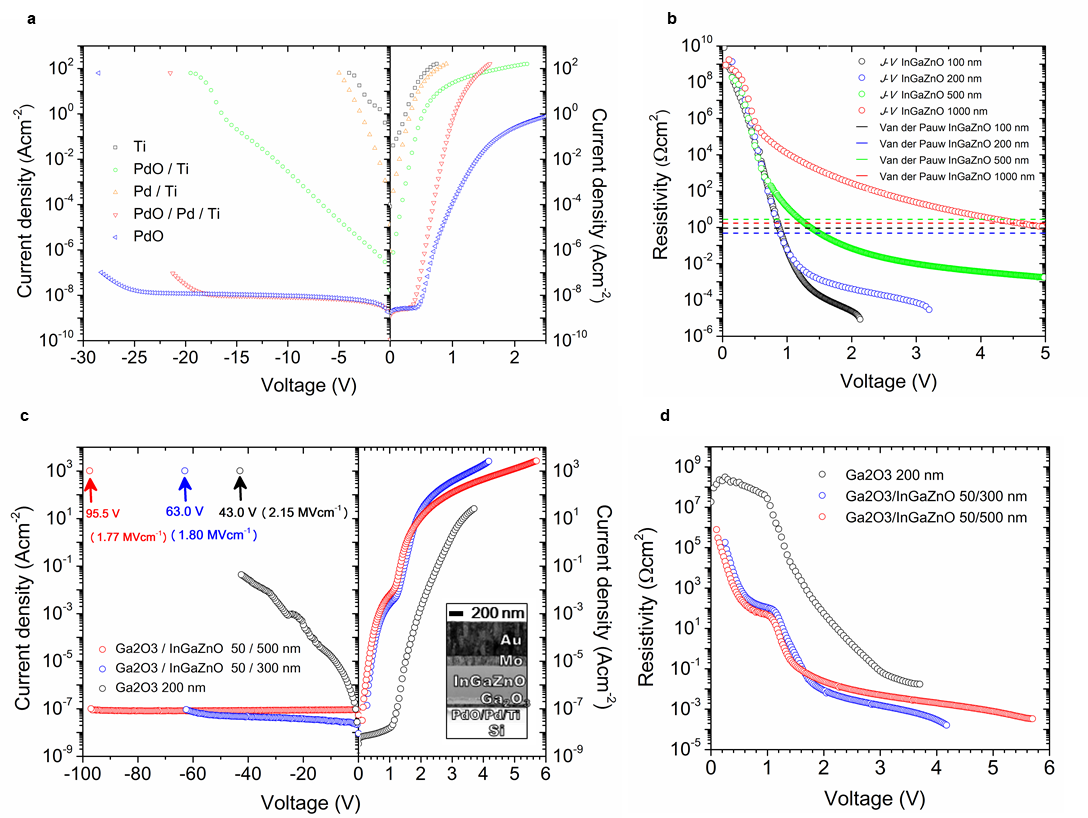
**

**Supplementary Fig. 1 | Current density (*J*)–voltage (*V*) and Resistivity (*R_on,sp_*)-voltage (*V*) characteristics. a,** *J-V* characteristics of 200 nm Schottky barrier diode (SBD) with the different metals for the optimization of Schottky electrode and contact metal. The legend represents the bottom electrode in Supplementary Table 1a. The thicknesses of the films and the fabrication process of the bottom electrode were the same for all samples. **b,** *R_on,sp_*-*V* characteristics obtained by differentiating the right-hand side of *J-V* characteristics in Fig. 3a**.** The dashed lines show the resistance obtained from the Van der Pauw measurement of the InGaZnO films on a glass substrate. **c,** *J-V* Characteristics of ATOP with Ga_2_O_3_ layer. Arrows indicate the breakdown voltage. Inset: Cross-sectional TEM image of the Ga_2_O_3_ (50 nm) / InGaZnO (500 nm) ATOP. **d,** *R_on,sp_*-*V* characteristics obtained by differentiating the right-hand side of *J-V* characteristics in (**c**).


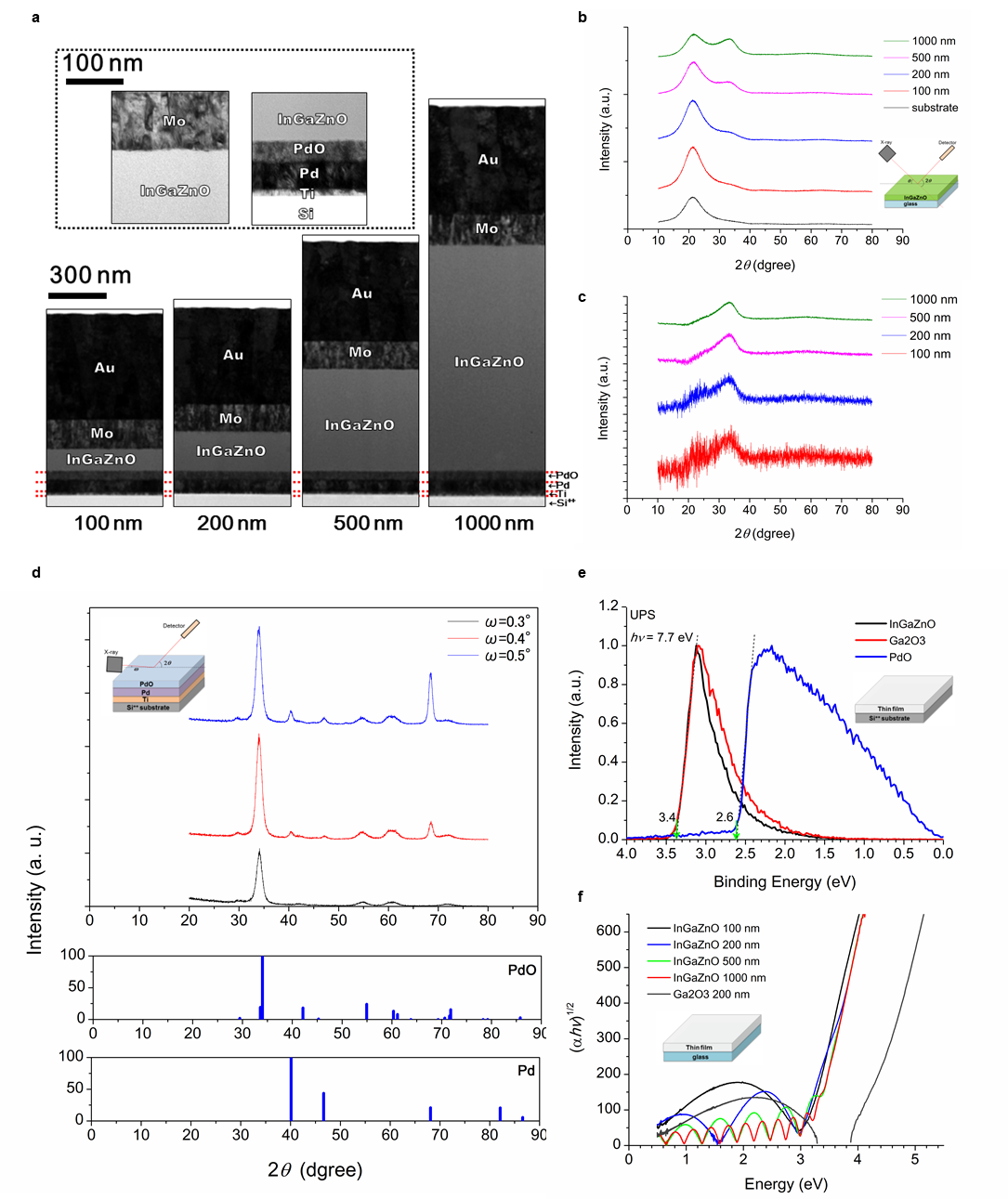


**Supplementary Fig. 2 | Analysis of thin-films. a,** Cross-sectional transmission electron microscope (TEM) images of the SBDs. The images in the dotted line area shows the electrode-InGaZnO interface (left) and the Schottky electrode-InGaZnO interface (right). **b,** The results of X-ray diffraction (XRD) measurements. XRD patterns of the InGaZnO thin films on quartz substrates for different thicknesses. **c,** Normalized XRD patterns of (**b**); the pattern from the substrate is subtracted, and the intensity is divided by the film thickness. These patterns are in good agreement with the reference patters in the XRD database, indicating an amorphous structure. **d,** Glancing angle XRD patterns of the Schottky electrode with PdO (40 nm) / Pd (50 nm) / Ti (15 nm) / Si^++^ (upper). The low incident X-ray angle *ω* shows a high surface sensitivity. The middle and the bottom patterns show PdO and Pd references of randomly oriented polycrystalline pattern. **e,** Ultraviolet photoelectron spectroscopy (UPS) spectra of the thin films of InGaZnO, Ga_2_O_3_ and PdO. **f,** UV-Vis transmission spectra of the thin films on quartz glass substrates.

**
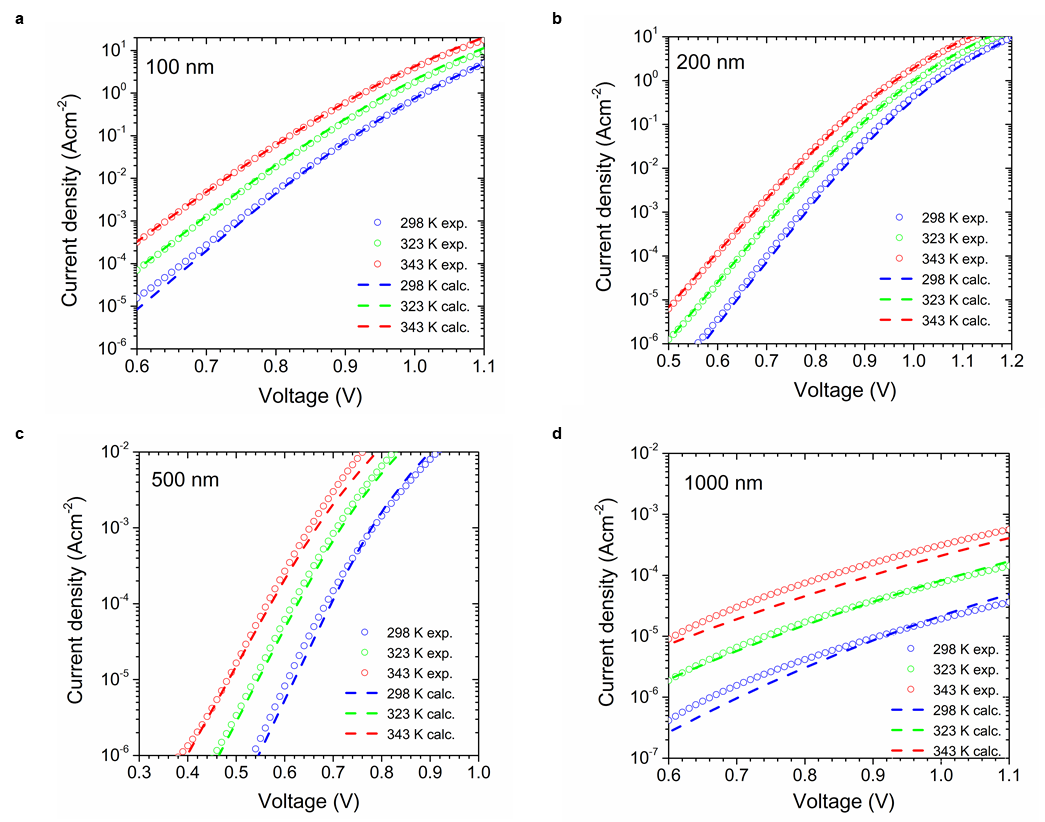
**

**Supplementary Fig. 3 | Comparison between the experimental results with the new Schottky-SCLC model at different temperatures.** InGaZnO SBDs with InGaZnO having thicknesses of (**a**) 100 nm, (**b**) 200 nm, (**c**) 500 nm and (**d**) 1000 nm. Description of the new Schottky-SCLC model and the parameters are shown in Method (See (6) New SCLC model) and Supplementary Table 1d, respectively.


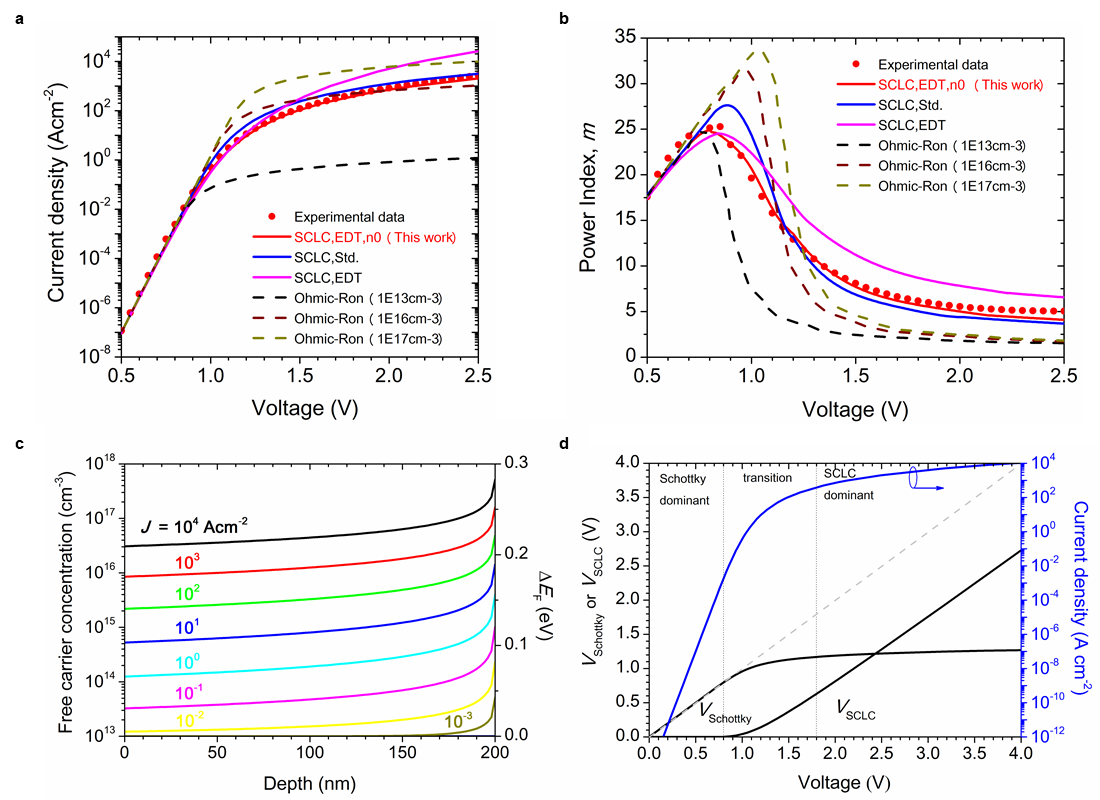


**Supplementary Fig. 4 | Calculated forward diode operation results on 200 nm InGaZnO SBDs with new Schottky-SCLC model. a,** Current density (*J*)-voltage (*V*) (See Equations (13), (14) and (24) in Method) and **b,** power law factor (*m*)*-V* characteristics (See Equation (11) and (12) in Method) for comparison with the different models. It is noted that *J_SCLC,EDT,n0_* of the new Schottky-SCLC model obeys *J_SCLC,Std._* of the ideal SCLC model for applied voltage over 2 V. **c,** Depth profiles of extrinsically injected free carrier concentration from the Ohmic electrode and band bending values of the quasi-Fermi level at each current density (See Equations (15)-(25) in Method). It is noted that the injected free carrier concentration is over 10^16^ cm^-3^ at any depth for *J* > 10^3^ Acm^-2^ (>2 V); this value is ~1000 times larger than that of the initial state (0 V). **d,** Distribution profile of the applied voltage at the Schottky interface (*V*_Schottky_) and in the semiconductor drift layer (*V*_SCLC_). Up to 0.8 V, most of the voltages (>90 %) were distributed at the Schottky interface. Over 1.8 V, most of the additional voltage was applied to the drift layer (See Equations (1), (3) and (24) in Method). All analyses in (**a**)-(**d**) used the fitting parameters of 200 nm InGaZnO in Supplementary Table 1d.

**
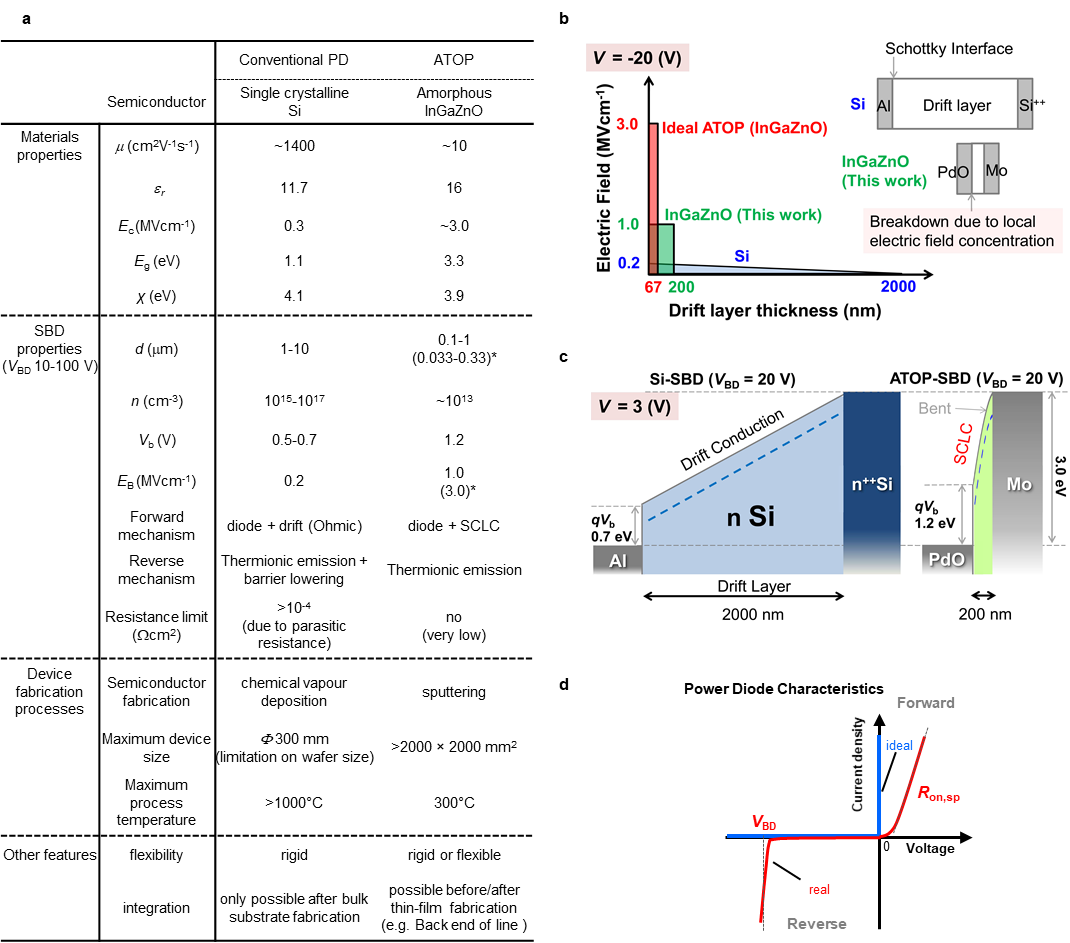
**

**Supplementary Fig. 5 | Comparison of characteristics between conventional power device (PD) and ATOP. a,** Table for the characteristics of single crystal Si and amorphous InGaZnO; the parameters obtained from references [1,18] and results in this work are summarized. **b,** The forward operation mechanism of the SBDs having breakdown voltage of 20 V: linear and nonlinear band diagrams observed in Si-SBD and ATOP-SBD, respectively. This band bending is due to the nonlinear SCLC conduction model, in which a high forward electric field is applied to the drift layer, which is much thinner for ATOP-SBDs than for Si-SBDs. **c,** The reverse operation mechanism of SBDs having breakdown voltage of 20 V: electric field profile at the voltage of -20 V. The integrated areas correspond to the breakdown voltage. As the critical electric field is an intrinsic value for each material, the thicknesses of drift layer can differ for SBDs having the same breakdown voltage. The shape of the characteristics (i.e. triangle and rectangle for Si and oxide semiconductors, respectively) is due to the initial carrier concentration. **d,** *J*-*V* characteristics of real and ideal power diodes.


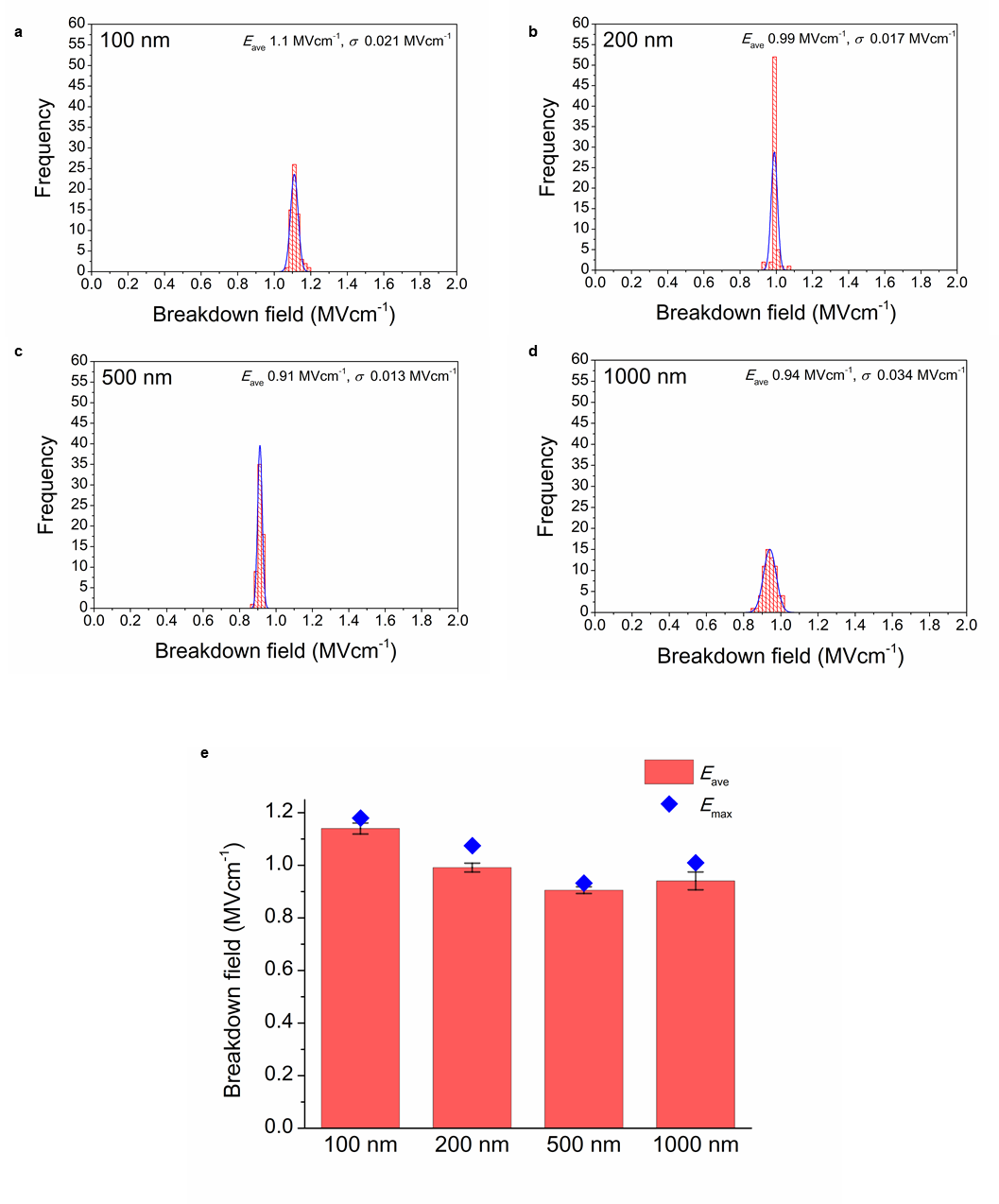


**Supplementary Fig. 6 | Breakdown behaviour of 64 SBDs.** Histograms of the breakdown fields of (**a**) 100 nm, (**b**) 200 nm, (**c**) 500 nm and (**d**) 1000 nm, and (**e**) bar chart of InGaZnO with various drift layer thicknesses. The height of the bars, the position of the blue squares and the error bars show the average breakdown field *E*_ave_, the maximum breakdown field *E*_max_ and the standard deviation *σ*, respectively.


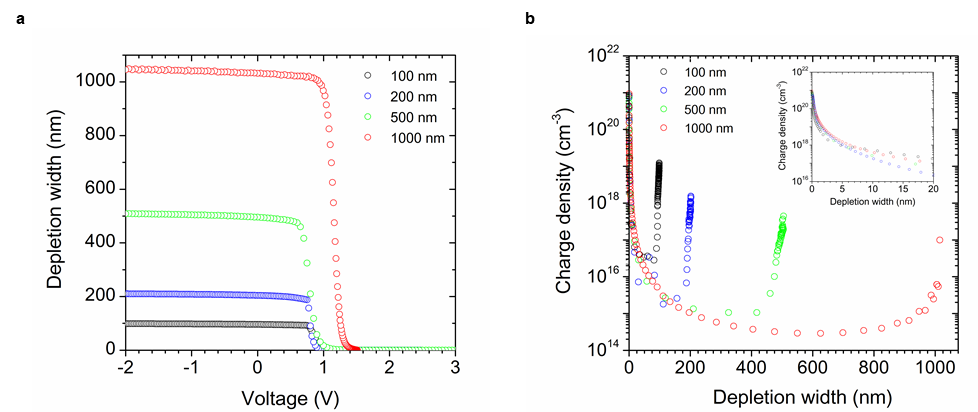


**Supplementary Fig. 7 | The results of capacitance(*C*)*-*voltage(*V*) measurements. a,** Depletion width (*W*)-*V* plots. *W* obtained from *C*-*V* characteristics of Fig. 3e through Equation (8) in Method. **b,** Depth profile of *N*_depl_ (the charge density contribution to *W*). *N*_depl_ obtained from 1/*C*^2^-*V* characteristics of Fig. 3e through Equation (9) in Method.


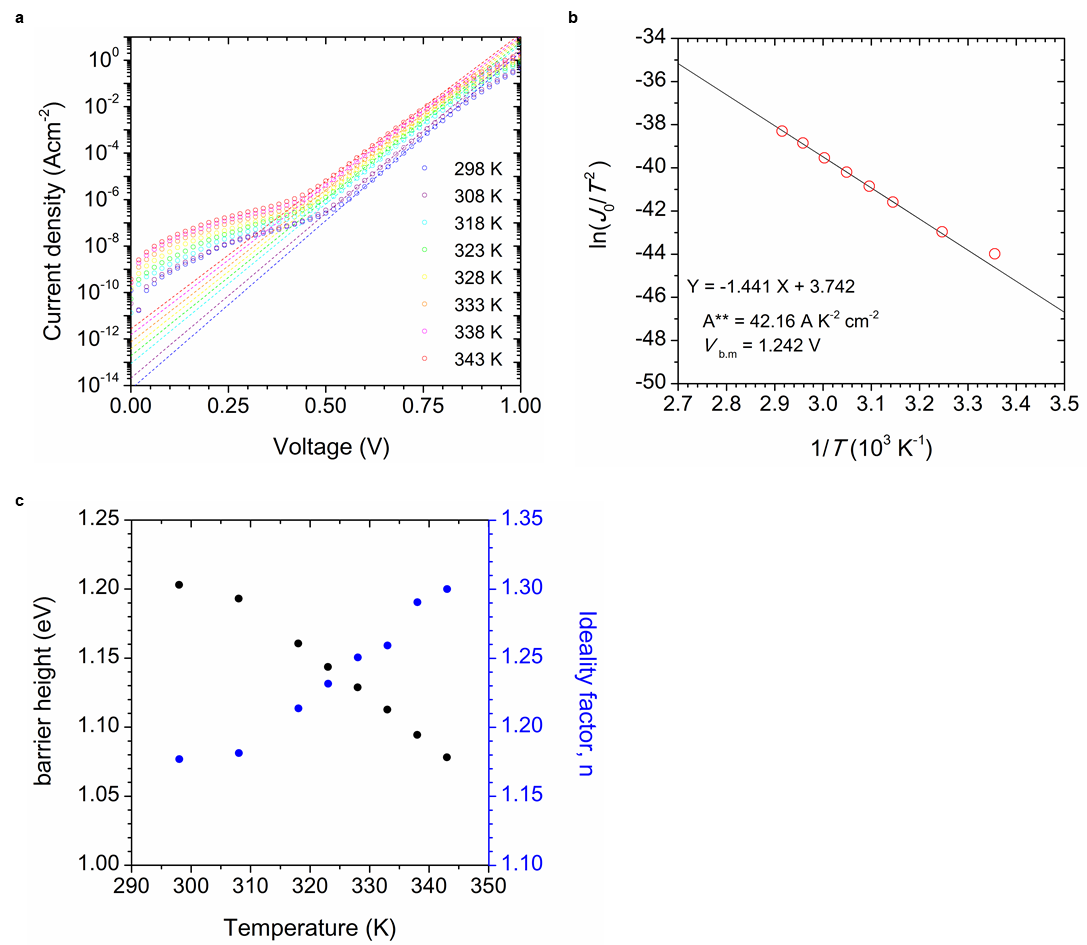


**Supplementary Fig. 8 | Temperature dependence of InGaZnO (200 nm) SBDs to obtain Richardson constant and Schottky barrier height. a,** Temperature dependence of *J-V* characteristics for saturation current *J*_0_ at each temperature. *J*_0_ is the intercept value of the dashed line according to the thermionic emission theory. **b,** Richardson plot for Richardson constant *A*^**^ and the mean barrier height *V*_B.M_. **c,** Temperature dependence of the Schottky barrier height and diode ideality factor n.

**Supplementary Table 1 | Parameters obtained in this work. a,** Diode parameters (from Fig. 3a and Supplementary Fig. 1). Voltages in parentheses show the current compliance voltage due to the measurement system. **b,** Parameters of the thin films on quartz glass substrates obtained from Hall measurement, UPS (from Supplementary Fig. 2e) and UV-Vis spectroscopy (from Supplementary Fig. 2f). The value with * is obtained from Reference [12]. **c,** Diode parameters obtained from *C-V* characteristics of the InGaZnO SBDs (from Fig. 3e and Supplementary Fig. 7). Values in parentheses in *W_depl_* column show the real drift layer thickness obtained by TEM. **d,** Fitted parameters used for the calculation in Supplementary Fig. 3.

**
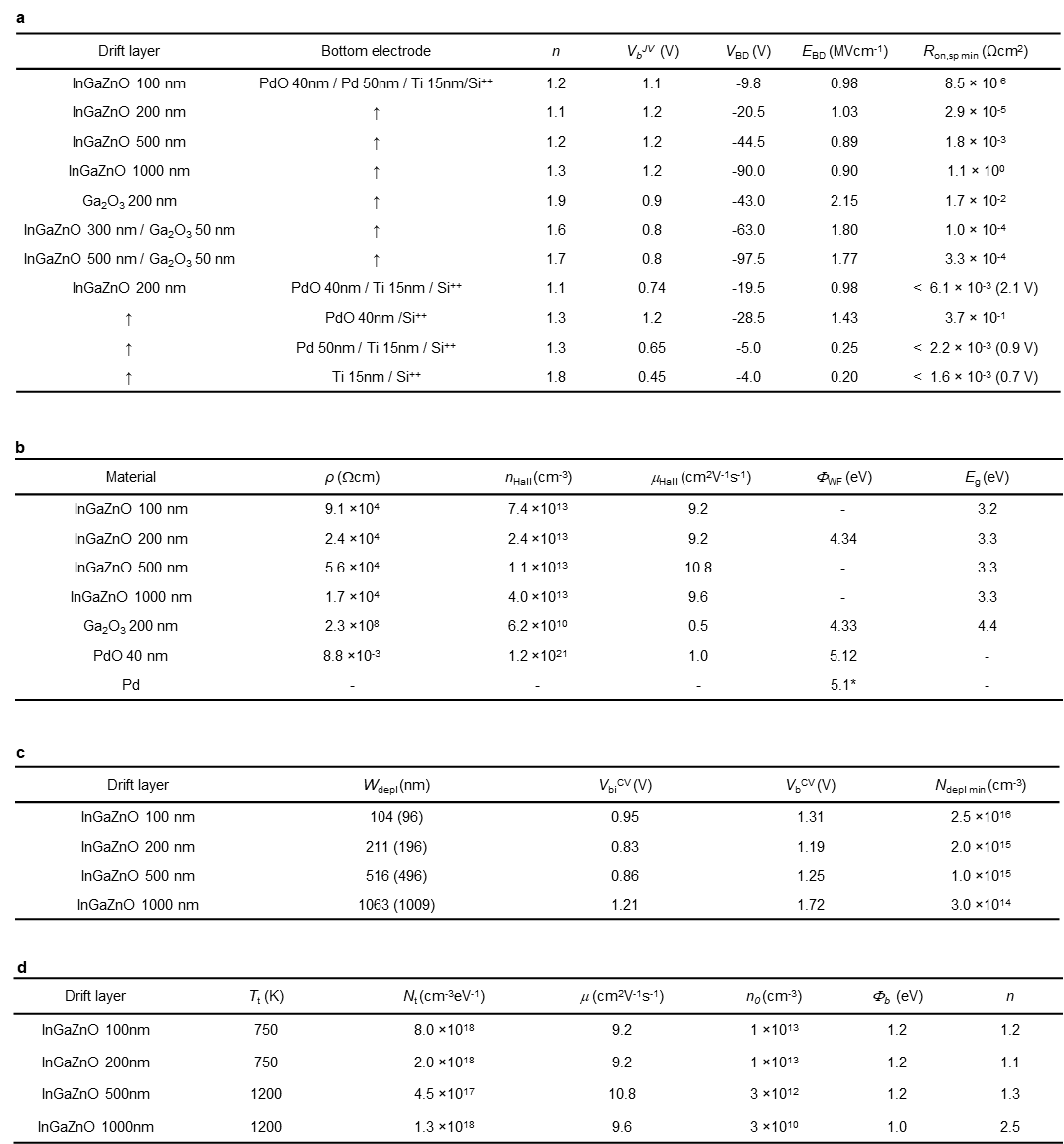
**
